# Possible Impact of 190G > A *CCR2* and Δ32 *CCR5* Mutations on Decrease of the HBV Vaccine Immunogenicity—A Preliminary Report

**DOI:** 10.3390/ijerph14020166

**Published:** 2017-02-08

**Authors:** Maria Ganczak, Karolina Skonieczna-Żydecka, Marzena Drozd-Dąbrowska, Grażyna Adler

**Affiliations:** 1 Department of Epidemiology and Management, Pomeranian Medical University, Szczecin 70-204, Poland; marzena.drozd@pum.edu.pl; 2 Department of Gerontobiology, Pomeranian Medical University, Szczecin 70-204, Poland; karolina.skonieczna-zydecka@pum.edu.pl (K.S.-Ż.); grazyna.adler@pum.edu.pl (G.A.)

**Keywords:** HBV, vaccination, immunogenicity, *CCR5*, *CCR2*, polymorphism

## Abstract

*Background*: Chemokine genetic variations are involved in infectious diseases such as hepatitis B virus (HBV). Several allelic variants might, in theory, affect the outcome of vaccination. *Objectives*: This study was carried out to examine the associations of Δ32 *CCR5* and 190G > A *CCR2* polymorphisms with a response to a primary course of three HBV vaccinations. *Methods*: Between December 2014 and December 2016, patients from three randomly selected primary care clinics in the West Pomeranian region (Poland), 1 month after receiving the third dose of HBV vaccine, were enrolled. Enzyme-linked immunosorbent assay (ELISA) system version 3.0 was used to detect anti-HBs and anti-HBc totals. The identification of polymorphisms were performed by a polymerase chain reaction technique using a single primer extension assay. Genotype distributions of responders versus non-responders to HBV vaccination were compared on the basis of anti-HBs level. *Results*: In 149 patients (mean age 60 years) the mean anti-HBs level was 652.2 ± 425.9 mIU/mL (range: 0–1111.0 mIU/mL). There were 14.1% (*n* = 21) non-responders to the HBV vaccine (anti-HBs < 10.0 mIU/mL). The wild type/Δ32 genotype of *CCR5* gene was found in 18.1% participants, and 1.3% were Δ32/Δ32 homozygotes. The frequency of allele A of the *CCR2* gene was 11.1%. Lower anti-HBs levels in Δ32/Δ32 homozygotes were observed (Me = 61 mIU/mL vs. Me = 660.2 mIU/mL; *p* = 0.048). As age was found to be a correlate to the anti-HBs titer (*r* = −0.218, *p* = 0.0075; 95% CI: −0.366–−0.059)—an analysis of a co-variance was performed which found a statistically significant (*p* = 0.04) difference in anti-HBs titres between Δ32/Δ32 homozygotes and other *CCR5* genotypes. The association between anti-HBs titres and *CCR2* genotypes was not statistically significant. *Conclusions*: Our study—which is a preliminary report that suggest this topic deserves further observation with larger sample sizes, different ethnicities, and other single nucleotide poly-morphisms (SNPs)—suggests the possible involvement of *CCR5* polymorphism in impairing the immunologic response to HBV vaccination, predominantly in relation to the passage of time.

## 1. Background

In humans, hepatitis B virus (HBV) is the most prevalent and the main infectious agent leading to liver disease. Viral hepatitis B (HB) continues to be a cause of considerable morbidity and mortality. The World Health Organization estimated that in 2012 around 240 million people were chronically infected with HBV worldwide, and approximately 780,000 die per year as a consequence of acute disease and chronic complications, such as cirrhosis and hepatocellular carcinoma. Globally, two billion people, more than a third of the world’s population, have been infected with HBV [1]. The prevalence of HBV in the Polish population has been found to be around 0.5%–1.5% [2].

Since 1981 HB has become a vaccine-preventable disease, due to safe and effective vaccines, typically given in a three-dose series [3]. In healthy individuals post-vaccine sero-conversion is as high as 90%–100% [4]. A concentration of anti-HBs 1–2 months after a primary series ≥10 mIU/mL is generally accepted as offering protection. Determinants that may decrease the immunogenicity include vaccine, host, and genetic factors [3,4,5].

Among genetic factors involved in host immune response to viral infection, chemokines and their receptors play a critical role; the most frequently studied being *CCR2* and *CCR5*, and their polymorphisms: Δ32 and 190G > A, respectively.

*CCR5* is a CC chemokine receptor, which influences the migration of granulocytes, macrophages, immature dendritic cells, CD8+ lymphocytes, Th1 lymphocytes and their activation. It is also a co-receptor for HIV entry into host cells [6,7]. The *CCR5* gene consists of a single coding exon, with non-functional allele containing a 32 bp deletion. A heterozygous (wt/Δ32) presents reduced levels of *CCR5* on the cell surface compared to a wild homozygous *CCR5* (wt/wt), while a homozygous mutant (Δ32/Δ32) presents higher levels of *CCR5* [6]. C-C chemokine receptor 2, is involved in modulating the immune response, as well as recruiting monocytes/macrophages to the sites of inflammation [8]. 

The polymorphism of the *CCR5* gene and DNA alterations might affect gene expression and then protein function, while the polymorphism G-A transition at position 190 in *CCR*2 gene, introducing a conservative change into the first transmembrane domain, is not associated with any clinical abnormality [9].

The frequency of allele Δ32 of *CCR5* gene varies from about 5% to 13% in different populations, and of allele A of *CCR2* gene from about 10% to 25% [10,11].

Chemokine genetic variations have been involved in infectious diseases such as HIV, HCV (Hepatitis C), HPV (Human papillomavirus), West Nile Virus, as well as HBV [12,13,14,15]. Previous studies suggested that the polymorphism 190G > A of the *CCR2* gene is associated with enhanced protection against HPV-16 infection [16]. Independent studies demonstrated the protective effect of *CCR5* Δ32 in recovery from a HBV infection, and provided genetic epidemiological evidence for the role of *CCR5* in the immune response to HBV [14,15]. However, the role of chemokines in HBV prevention has not been fully clarified [17]. Several allelic variants might, in theory, affect the outcome of vaccination. Nevertheless, data on the relationship of both Δ32 *CCR5* and 190G > A *CCR2* polymorphisms and the HBV vaccine immunogenicity are sparse.

## 2. Objectives

The study objective was to examine Δ32 *CCR5* and 190G > A *CCR2* polymorphisms in the context of HBV vaccination response.

## 3. Methods

### 3.1. Setting and Sampling

The study was conducted between December 2014 and December 2016 among 185 consecutive, unrelated adult patients presenting one month after taking a third HBV vaccination dose, at three randomly selected urban primary care clinics (PCCs) located in Szczecin, in the West Pomeranian region of Poland. Patients with serological markers of HBV infection (anti-HBc total) were excluded from the study (*n* = 24; 12.5%). Among those remaining (*n* = 161), the extraction of genome DNA failed in 12 (7.5%), therefore 149 individuals were finally analysed.

### 3.2. Study Instrument

After signing informed consent forms, participants filled out a questionnaire which anonymously queried them on their demographics (age, gender, weight and height), smoking habit and medical history of HB.

### 3.3. Sero-Testing and Genetic Testing

Blood samples were collected by venipuncture. Enzyme immunosorbent assay (ELISA) system version 3.0 (Abbott Laboratories Inc., Abbott Park, IL, USA) was used to detect anti-HBs and anti-HBc total.

Genomic DNA from leukocytes of peripheral blood, collected into sterile tubes containing ethylene diamine tetraacetic acid (EDTA) solution was isolated with the use the QIAamp DNA extraction kit (Qiagen, Hilden, Germany). The extraction followed manufacturer instructions; DNA samples were stored at 4 °C. Subsequently, genotypes were determined by a polymerase chain reaction (PCR) technique, according to a previously described protocol with the following temperature profile: the initial denaturation for *CCR2* was 94 °C for 5 min; 38 cycles of 20 s at 94 °C, 40 s at 57 °C and 40 s at 72 °C; for *CCR5* the initial denaturation was 94 °C for 5 min; 38 cycles of 30 s at 94 °C, 30 s at 60 °C and 45 s at 72 °C; the final extension step was 72 °C for 7 min for each of the polymorphisms [18,19]. For the analysis of *CCR2* and Δ32 *CCR5* mutations, a sequence specific PCR was run using the following two primer pairs: forward 5′-TTG TGG GCA ACA TGA TGG-3′ and reverse 5′-GCA TTC CCA AAG ACC CAC TC-3′ and forward 5′-GAT AGG TAC CTG GCT GTC CAT-3′ and reverse 5′-ACC AGC CCC AAG ATG ACT ATC T-3′, respectively (TIB MOL BIOL, Poznań, Poland). After of the use of a *BsaBI* restriction enzyme (Thermo Fisher Scientific, Waltham, MA, USA), a wild allele (G) *CCR2* gene was detected as a 163 bp fragment, while a mutant allele (A) 145 and 18 bp, and wild (wt) and mutant (∆32) alleles of *CCR5* gene were detected as 242 and 210 bp fragments, respectively. For quality control purposes, results were verified by performing re-genotyping of randomly selected samples; all results were reliable and replicable. To assess possible differences in the frequencies of ∆32 and 190G > A polymorphisms, a control group from the HAPMAP database was used [20]. The study was approved by the institutional ethics committee (KB-0012/180/13).

### 3.4. Statistical Analyses

Mann-Whitney/*t*-student tests were used for the comparisons of continuous variables, a χ^2^ test to verify whether genotype and allele frequencies matched the Hardy-Weinberg equilibrium and to assess whether there are differences in genotype and alleles distribution between vaccine responders/non-responders. Genotype comparisons by means of anti-HBs values were performed using an ANOVA test, and nonparametric counterparts (Kruskall-Wallis, Mann-Whitney), as appropriate. The level of anti-HBs is influenced by gender, smoking and BMI (Body Mass Index) [3,5], so analyses of variance were initially performed separately for each and then together to determine any interactions between variables. To establish the OR and 95% CI logistic regression was used. The significance level of the test was set to 0.05. Statistical analyses were performed using the StatView version 5.0 software (BrainPower Inc., Calabasas, CA, USA).

## 4. Results

Patient demographic characteristics are shown in Table 1.

### 4.1. Genotyping

The following *CCR5* Δ32 genotype frequencies were assessed: wild type/wild type (wt/wt): 80.6% (*n* = 120), wt/Δ32: 18.1% (*n* = 27), Δ32/Δ32: 1.3% (*n* = 2).

Regarding *CCR*2 polymorphism the following genotype frequencies were identified: GG: 117 (78.5%), GA: 31 (20.8%), AA: 1 (0.8%). The major allele frequency was 88.9%.

The distribution of the Δ32 *CCR5* and gene *CCR*2 polymorphisms followed the Hardy-Weinberg equilibrium (χ^2^ = 0.1605; *p* = 0.73 and χ^2^ = 0.4714; *p* = 0.49, respectively).

There were no differences in the frequencies of studied polymorphisms between the study group and HAPMAP controls (*CCR5*: *p* = 0.34, *CCR2*: *p* = 0.81 respectively).

### 4.2. Anti-HBs Titres

**Figure 1:**
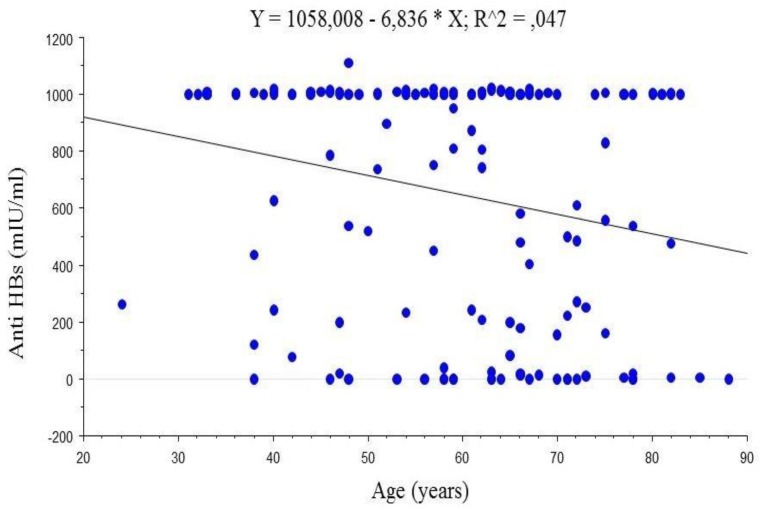
Correlation of anti-HBs titres after receiving the third dose of hepatitis B virus (HBV) vaccine with patient age (*n* = 149).

The median anti-HBs titre for the study participants was 652 ± 4259 mIU/mL (range: 0–1111.0 mIU/mL). There were 21 (14.1%) non-responders to the HBV vaccine (anti-HBs < 10.0 mIU/mL) for whom the median anti-HBs titre was 0.0 mIU/mL. For 128 (85.9%) responders (anti-HBs ≥ 10.0 mIU/mL) the median anti-HBs titre was 1001.0 mIU/mL. There was a statistically significant difference in anti-HBs titres between the two groups (*p* < 0.0001). In women the median anti-HBs titre was 1001 mIU/mL, in men 772.0 mIU/mL (*p* = 0.08). Anti-HBs titres were negatively correlated with patient age (*r* = −0.218, *p* = 0.0075; 95% CI: −0.366–−0.059); Figure 1. No correlation was found regarding BMI values and anti-HBs titre (*r* = −0.019; *p* = 0.82, 95% CI: −0.179–−0.142), or associations regarding smoking status, gender and anti-HBs level (*p* = 0.12; *p* = 0.06, respectively).

### 4.3. Associations between CCR2 and CCR5 Genotypes and Anti-HBs Titres

In 21 non-responders medians of anti-HBs titres were 0.0 mIU/mL despite the *CCR5* genotype; *p* = 0.95. In 128 responders medians of anti-HBs titres were 1001.0 mIU/mL for individuals presenting wt/wt and Δ32/wt genotypes, and 122.0 mIU/mL for those presenting Δ32/Δ32 genotype; *p* = 0.273. The median of anti-HBs titres in *CCR2* GG homozygotes was 1001 mIU/mL, in GA heterozygotes 1001 mIU/mL and in the single AA homozygote 1001 mIU/mL.

A one-way ANOVA revealed a statistically significant association between *CCR5* genotype in a co-dominant model of inheritance and anti-HBs titres (*p* = 0.04). Therefore, we adapted a recessive model of inheritance for a Δ32 *CCR*5 polymorphism. The median anti-HBs titre in Δ32/Δ32 homozygotes was 61 mIU/mL and in other genotypes: 660.2 mIU/mL (*p* = 0.0478).

Due to the lack of normal distribution in anti-HBs titre values, non-parametric tests were also performed. The analysis did not show statistical significance regarding the association between anti-HBs titres and the co-dominant model of Δ32 *CCR5* model of inheritance (*p* = 0.16). However, a statistical tendency towards lower anti-HBs titres in Δ32/Δ32 homozygotes was confirmed in a recessive model (*p* = 0.078).

**Figure 2:**
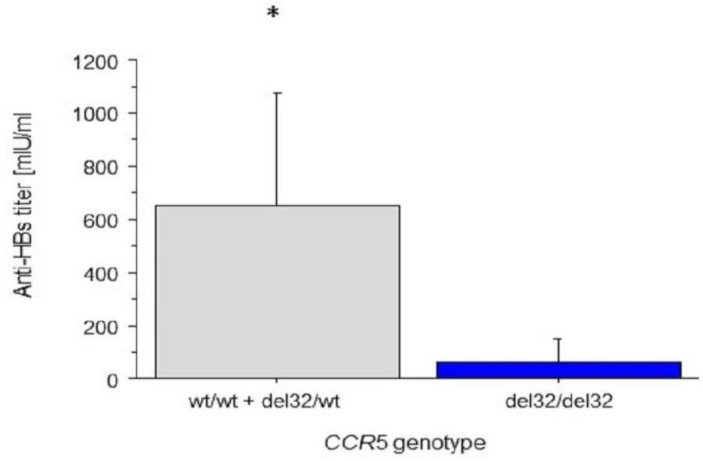
Anti-HBs titres by *CCR*5 recessive genotype and age as a cofactor (* *p* = 0.04; bars indicate standard deviations); *n* = 149.

Age, gender, BMI and smoking status were taken into consideration for further analyses when appropriate. As age was found to be a correlate to the anti-HBs titre the corresponding analysis of a co-variance was performed. A difference in anti-HBs titres between Δ32/Δ32 homozygotes and other *CCR5* genotypes was found to be statistically significant (*p* = 0.04); Figure 2. 

Additionally, two-way ANOVA for *CCR*5 recessive genotype and smoking as independent variables was performed; no significant results for the main effects of the analysis (*CCR*5: *p* = 0.07; smoking: *p* = 0.97) and interactions (*CCR*5 * smoking: *p* = 0.66) were found. The OR of the Δ32 *CCR*5 gene polymorphism in recessive manner towards real anti-HBs titres/vaccine response was found to not be statistically significant (*p* = 0.265, *p* = 0.197, respectively).

No association between *CCR2* genotype and anti-HBs titres were found in one-way ANOVA analyses in co-dominant, dominant, recessive and over-dominant models of inheritance (*p* > 0.05, *p* = 0.33, *p* = 0.41, *p* = 0.42, respectively). Nonparametric analyses in *CCR2* genotype towards ant-HBS tires was confirmed to be nonsignificant in all models of inheritance (*p* = 0.45, *p* = 0.21, *p* = 0.69, *p* = 0.24, respectively).

Finally, post hoc power analyses using G * Power [21] computer software was performed. This is a flexible statistical power analysis program for social, behavioural, and biomedical sciences. Statistical powers analyses observed were under the recommended 0.8 level [22], thus to obtain larger effects, the sample size would have to be increased.

## 5. Discussion

### 5.1. Results Overview

Of the 149 patients, presenting at PCCs one month after receiving the third dose of HBV vaccine, 12.5% were not protected against HBV infection. A wild type/Δ32 genotype was found in 18.1% participants, 1.3% were Δ32/Δ32 homozygotes. The frequency of allele A of *CCR2* gene was 11.1%. Age was found to be a correlate to the anti-HBs titre. When adjusted to age, the difference in anti-HBs titres between Δ32/Δ32 homozygotes and other *CCR5* genotypes was statistically significant. No statistically significant association between anti-HBs titres and *CCR2* genotypes was found.

### 5.2. Anti-HBs Titres

In patients who completed a 3-dose HBV vaccination schedule, anti-HBs titres were negatively correlated with age. Decreasing responses to immunization with advanced age have been described previously [5,23]. A model on sero-protection rates presented by Van Der Meeren et al. showed a statistically significant decrease in anti-HBs sero-protection rate with age, and predicted that it remains ≥80% up to 60 years of age [23]. This is in line with our results: 83% of patients from PCCs, with the mean age of 60 years, gained sero-protection after HBV vaccination. Although some other studies reported correlations between anti-HBs levels after HBV vaccination and host factors, such as male gender, obesity, and smoking [3,5,23], similarly to Williams et al. [24] we did not find such correlations, possibly due to the limited sample size.

### 5.3. Frequencies of Δ32 CCR5 and 190G > A CCR2 Allele

According to medical literature, the mean frequency of Δ32 *CCR5* allele in Europe is approximately 10%, with the highest allele frequency (12%) observed among Nordic populations and the lowest (5%) in the regions of Southeast Mediterranean [10]. In Poland Δ32 *CCR5* allele frequency is comparable to that found in Caucasian populations and follows the pattern of the north-southern gradient observed in Europe. The lowest frequencies of Δ32 *CCR5* were detected in provinces in central and north-western regions of the country [11]. The frequency of the Δ32 allele among PCCs patients followed the pattern of the north-southern gradient observed in Europe [10] and was similar to that reported in orthopaedic trauma patients and staff [25], as well as newborns [26] from regional hospitals and data reported among Polish blood donors [11]. Moreover, there were no differences in the frequencies of both studied polymorphisms between the study group and HAPMAP controls [20].

### 5.4. The Association between Anti-HBs Titres and CCR5 and CCR2 Genotypes

Chemokines are responsible for innate and adaptive immune responses and surveillance [27]. The variation in chemokine release is individual and linked to polymorphisms of genes. Only a small number of studies—involving animals and humans—have been carried out regarding the involvement of chemokines in response to infectious agents [28,29,30,31,32]. As an example, Algood et al. tested the consequences of Δ32/Δ32 genotype in mice and found a more robust T-cell response to several infectious agents [31]. The authors observed more dendritic cells in lymph nodes and increased pulmonary inflammation, due to a greater T-cell response in *Mycobacterium tuberculosis* infection in mutant (Δ32/Δ32) mice, when compared to wt/wt mice. 

Given the importance of cell response in HBV infection, including a finding that the production of a non-functional *CCR5* (Δ32 *CCR5*) increases the likelihood of recovery from HB in humans [11,12,33], and given the increased T-cell response to various antigens observed in animal models [31], we conducted the first study on the human population and hypothesized that both homozygous Δ32/Δ32 of *CCR5* gene and homozygous AA of *CCR2* gene might be bio-markers for immunological response in subjects after a HBV vaccination. This hypothesis was tested by genotyping *CCR2* and *CCR5* genes for two SNPs (190G > A *CCR2* and Δ32 *CCR5*) in a group of Polish patients in the context of response to HBV vaccination. 

Age was found to be a negative correlate to the anti-HBs titre. The median anti-HBs titre in Δ32/Δ32 homozygotes was more than ten times lower than observed in patients with other genotypes, this was a statistically significant difference. Though, it might be hypothesized that *CCR5* mutation can influence the outcome of HBV immunization, i.e., be associated with impaired response to vaccination with the passage of time. 

However, vaccine response is a complex issue with multiple factors involved, including genetic variations. Furthermore, genetic interactions are also complex, therefore it is unlikely that a single allelic variants investigation would be enough to show their dominant role in impaired immunologic response after HBV vaccination. If a singular SNP was considered as an independent risk factor toward impaired vaccine response, it may an extended period to observe. This might explain why the impact of a bi-allelic polymorphism is best observed in older age. Over the years, most environmental factors (e.g., obesity, smoking status) begin to be set at a more constant level, the direct impact of a genetic variation on the selected clinical parameter can be seen more clearly. 

This preliminary evidence needs further study on the role genetic factors play in the immune response to the vaccine, including a decrease in vaccine immunogenicity. Thorough medical literature search was unsuccessful in finding previous studies related to this subject, therefore comparisons with other surveys are not presented in this paper. Any consideration on the clinical utility of our findings should include a question of *CCR5* polymorphism as one of the possible targets for the screening of HBV vaccination effectiveness. 

## 6. Limitations

A clear advantage of the current study is its pioneering character—to our knowledge this is the first study on the human population which examined Δ32 *CCR5* and 190G > A *CCR2* polymorphisms in the context of a response to HBV vaccination. Moreover, the study was conducted among patients from randomly selected PCCs, therefore the study population may be representative of the whole region. In addition, the frequencies of both studied polymorphisms are comparable to those found in Caucasian populations [7,8,16].

The main limitations of this study were the small sample size of subjects (including the number of individuals in the group with Δ32/Δ32), recruited from the one region, and involving one ethnic group. However, due to the low prevalence of Δ32/Δ32 *CCR5* homozygotes in the Caucasian population (less than 1% [11]), collecting samples from homozygotes vaccinated for HBV with 3 doses, who have not been infected with HBV previously, was difficult. These limitations made our sample ever smaller. Therefore, data are presented as a preliminary report, and the conclusions that can be drawn are limited from such low numbers. 

Due to these limitations, which may affect the results, we recommend further studies in patients presenting one month after taking the third HBV vaccination dose, with a larger sample size and different ethnicities, to validate our results. Also, due to the interference of other cytokines in immunologic response after HBV vaccination, the effect of other allelic variants should also be investigated. Therefore, we recommend further studies related to this issue.

## 7. Conclusions

Our study—which is a preliminary report that suggest the topic deserves further observation—underscores the possible involvement of *CCR5* polymorphism in impairing the immunologic response to HBV vaccination, predominantly in relation to the passage of time. Additional studies are necessary to validate our findings, as well as to clarify the clinical utility of *CCR5* polymorphism. This paper may contribute to further anthropological and epidemiological surveys on the subject. 

## Figures and Tables

**Table 1:** Study group characteristics * (*n* = 149).

Variable	All (*n* = 149)	Women (*n* = 85)	Men (*n* = 64)	p **
Age (years)	59.4 ± 13.6	61.0 ± 13.8	57.2 ± 13.0	0.08
Body mass (kg)	76.7	68.0	84.5	<0.0001
Height (cm)	167.4 ± 8.5	162.5 ± 6.4	173.9 ± 6.3	<0.0001
BMI (kg/m^2^)	26.7	26.1	27.9	0.06

* ex re variables that followed normal distribution data are shown as mean ± standard deviation; for variables without normal distribution characteristics are expressed as median. ** women vs. men. BMI: Body Mass Index.
